# *In-vitro* Antimycoplasmal Activity of Triclosan in Combination with Fluoroquinolones against Five *mycoplasma* Species 

**Published:** 2012

**Authors:** Lei Li, Weimin Shen, Kaiyu Zhang, Xudong Tang, Na Guo, Fengge Shen, Mingxun Xing, Lihui Liua, Peng Yuan, Qiyun Shi, Junchao Liang, Lu Yu

**Affiliations:** a *Key Laboratory of Zoonosis Research, Ministry of Education, Institute of Zoonosis, College of Animal Science and Veterinary Medicine, Jilin University, Changchun 130062, China. *; b *Department of Infectious Diseases, First Hospital of Jilin University, Changchun 130021, P.R. China.*; c *Key Lab for New Drug Research of TCM, Research Institute of Tsinghua University in Shenzhen, Shenzhen 518057, P.R. China. *; d *Department of Food Quality and Safety, College of Quartermaster Technology, Jilin University, Changchun 130062, China. *; e *State Key Laboratory for Molecular Virology and Genetic Engineering, Chinese Center for Disease Control and Prevention, Beijing 100176, China. *

**Keywords:** Mycoplasma, Triclosan, Fluoroquinolones, Susceptibility, Combination

## Abstract

Mycoplasmosis caused by mycoplasma has immensely reduced the performance of commercial animal husbandry, along with prevalence and increase of drug resistance in mycoplasma, thus new agents and therapies are urgently needed. Triclosan is a broad spectrum antimicrobial agent with a favorable safety profile. In the present study, we tested the antimycoplasmal activity of triclosan alone, as well as the *in-vitro *interaction of triclosan and the fluoroquinolones, gatifloxacin (GAT), moxifloxacin (MXF), levofloxacin (LVX), sparfloxacin (SPX), ciprofloxacin (CIP), enrofloxacin (EFX), and norfloxacin (NOR), against five mycoplasma species. This study demonstrated that triclosan had antimycoplasmal activity against both fluoroquinolones-sensitive species and a fluoroquinolones-resistant species isolated from clinic, with minimum inhibitory concentrations (MICs) of 16.0-64.0 μg/mL and 64.0 μg/ mL, respectively. A synergistic antimycoplasmal effect between triclosan and GAT, MFX or EFX against the five mycoplasma species was observed, with modulation factors (MFs) of 4-8, 4-16, 8-32, respectively, and fractional inhibitory concentration indexes (FICIs) of 0.375- 0.500, 0.350-0.500, 0.281-0.375, respectively. The combination of triclosan with LVX, SPX, CIP or NOR displayed either synergistic activity or indifference against the same mycoplasma species with MFs of 2-64, 4-16, 2-16, 2-64, respectively, while FICI values range from 0.516- 0.750, 0.500-0.625, 0.306-0.750, and 0.615-0.750, respectively. No antagonism was observed for any drug combination against any of the species tested. To the best of our knowledge, this is the first report that triclosan has synergistic activity with fluoroquinolones against mycoplasma species.

## Introduction

*Mycoplasma *is a wall-less microorganism (1) that can cause serious livestock diseases such as contagious bovine and caprine pleuropneumonia, contagious agalactia in small ruminants, calf pneumonia, enzootic pneumonia in pigs and chronic respiratory disease in poultry (2). *Mycoplasma bovis *(*M. bovis*) is a major, worldwide pathogen that is often overlooked. It causes respiratory disease, mastitis, and arthritis in cattle (3). The prevalence of family farms seropositive for *Mycoplasma gallisepticum *(*M. gallisepticum*) and *Mycoplasma synoviae *was greater than 50%, even reaching 100% in most tested counties in Argentina, which presents a high risk to commercial poultry production (4). Contagious caprine pleuropneumonia, a respiratory mycoplasmosis caused by *Mycoplasma mycoides subsp. capri *(*M. mycoides subsp. capri*), causes high-mortality outbreaks in goats (5). Thus, mycoplasma infections have been shown to reduce the performance of commercial livestock, like egg-laying hens, dairy cows and goats, leading to significant economic loss (6).

Similar to all mollicutes, mycoplasma is inherently refractory to certain groups of antibiotics owing to its lack of a cell wall. Furthermore, evidence has shown that the mycoplasma genus is resistant to antibiotics traditionally used for their control, including tetracycline, tilmicosin and spectinomycin (3). In contrast, fluoroquinolones, with excellent pharmacokinetic profiles and activity against both Gram-negative and Gram-positive organisms, have been developed (7) and can be used to control mycoplasmosis. However, in field conditions, fluoroquinolones treatment cannot eradicate infections (8). Therefore, we must explore new alternatives to effectively target mycoplasma infections.

Triclosan (2,4,4’-trichloro-2’-hydroxydiphenyl ether) is a non-ionic broad-spectrum antimicrobial agent that has been extensively used in deodorants, soaps and other dermatological preparations due to its favorable safety profile (9,10). The efficacy of triclosan as an antibacterial agent, in both systemic and topical applications for various infections, shows great promise and excellent safety (11). In addition, triclosan has demonstrated antiplaque, antigingivitis, antibacterial, and anti-inflammatory (12-14) properties (15). Additionally, there have been no reports of triclosan-resistant microbes in the wild (16).

In this study, we examined the effect of fluoroquinolones treatment, at different dosages, on the persistence of mycoplasma species tested. We also evaluated the antimycoplasmal activity of triclosan as a candidate for preventive and therapeutic uses. We further investigated the interactions of triclosan in combination with the first-line fluoroquinolone drugs, including gatifloxacin (GAT), moxifloxacin (MXF), levofloxacin (LVX), sparfloxacin (SPX), ciprofloxacin (CIP), enrofloxacin (EFX) and norfloxacin (NOR) against five veterinary mycoplasma species using a checkerboard microdilution assay.

## Experimental

*Reagents and antibiotics*

Triclosan and dimethyl sulfoxide (DMSO) were purchased from Sigma-Aldrich (Sigma, St. Louis, MO). Triclosan was dissolved in DMSO at a concentration of 5 g/L under sterile conditions and stored at -70°C until used. Seven fluoroquinolones, GAT, MXF, LVX, SPX, CIP, ENR, and NOR, were purchased from the National Institute of the Control of Pharmaceutical and Biological Products, Beijing, China. MXF was dissolved in water. The other fluoroquinolones were prepared in 0.1 M NaOH.

*Microorganisms and growth conditions*

*M. mycoides subsp. Capri *Y-goat, *M. gallisepticum *BG44T, *M. gallisepticum *PG31 and *M. bovis *PG1 were obtained from the China Medical Culture Collection Center (CMCC). *M. bovis *8421, a clinical isolate, was obtained from the Animal Hospital of Jilin University, Changchun, China. *M. bovis *PG1 and *M. bovis *8421 were grown in PPLO broth (Difco) (17) containing fresh yeast extract, tryptone, 20% horse serum, 0.5% glucose, and 500 U/mL penicillin G. Using the same method, *M. gallisepticum *and *M. mycoides subsp. Capri *Y-goat were inoculated into MEM broth (Invitrogen) (18) formulated with 20% horse serum, and 500 U/mL penicillin G. Meanwhile, phenol red, at a final concentration of 0.0004% (wt/vol), was supplemented as a color redox indicator for all species. Then, all tested mycoplasma species were maintained at 37°C under 5% CO2 in an incubator (Thermo) for 2-3 days until their color changed from pink (pH 7.6~7.8) to orange-yellow (pH = 6.8) visually.

The color-changing units (CCU) per mL of mycoplasma were determined by using a ten-fold serial dilution method. The CCU was evaluated by examining the order of bottle with color changing. For example, the color of the 10th bottle was not changed; however, the color of the 1st through 9th bottles was changed from pink to orange-yellow. Thus, it demonstrated that the concentration of the first bottle was approximately 108 CCU/mL (18). Hence, we could obtain the concentration we need using this serial dilution method.

*In-vitro antimycoplasmal susceptibility testing*

Conventional *in-vitro *antimycoplasmal susceptibility testing was performed using the broth dilution method on 96-well microtiter plates, in accordance with the guidelines for minimum inhibitory concentration (MICs) testing against veterinary mycoplasma species (19). Briefly, serial two-fold dilutions of antimicrobial agents were individually prepared in mediums to obtain the required concentrations. Then, these samples were added to the respective columns. Each well in columns #1-10 contained 100 μL of the medium with the agent (columns #1-9), or with no agent (column #10) as a growth control. Each of the wells was added to 100 μL of mycoplasma culture containing about 103~105 CCU/mL organisms in columns #1-10. Each well in columns #11 and #12 contained 200 μL of medium adjusted to a pH of 6.8 (pH control) or uninoculated growth medium (sterility control), respectively (20). Plates were sealed, incubated at 37°C and inspected daily for color change until the color in the growth well matched that of the end-point control (orange-yellow). Since the fluoroquinolones stock solution contained 0.1 M NaOH and triclosan contained DMSO that comprised < 1% of the total test volume, a control was carried out to determine if the presence of traces of compound affected the pH of the medium or had an inhibitory effect on the growth of mycoplasma species, respectively.

The MIC was defined as the lowest drug concentration that showed no color change in the medium. Experiments were performed in triplicate, and the median MIC value was calculated.

*Checkerboard titration for drug combination studies*

The effects of triclosan and fluoroquinolones combinations against each strain were determined by using the checkerboard microdilution method on 96-well microtiter plates, as previously described (21). The checkerboard plates were inoculated with 103~105 CCU/mL of microorganisms in each well, and the final concentrations of both drugs (two-fold dilutions) ranged from 1/32 to 4 times the MIC for triclosan and from 1/256 to 4 times the MIC for fluoroquinolones. After the inoculation and agitation, the microplates were incubated at 37°C until a color change was appreciated. The MICs alone, or in combination, were read as described above (22). Each isolate was tested in triplicate on different days.

To analyze the interaction between triclosan and fluoroquinolones, we employed two models: a modulation factor (MF) and a fractional inhibitory concentration index (FICI). An MF was used to express the modulating effects of triclosan on the MICs of fluoroquinolones. The formula used was MF = MIC (fluoroquinolones)/MIC (fluoroquinolones + modulator) (23). The fractional inhibitory concentration index (FICI) is most frequently used to describe the drug interactions. The FICI, for a combination of two antimicrobials, was calculated according to the following equation:

FICI = FICA + FICB = (A/MICA) + (B/MICB)

Here, A and B are the MICs of drugs A and B in combination, respectively, and MICA and MICB are the MICs of drugs A and B when acting alone, respectively. The interpretation of the FICI value was as follows: an FICI ≤ 0.5 demonstrated synergy, an FICI between 0.5 and 

## Results

*Determination of drugs MICs*

**Figure 1:**
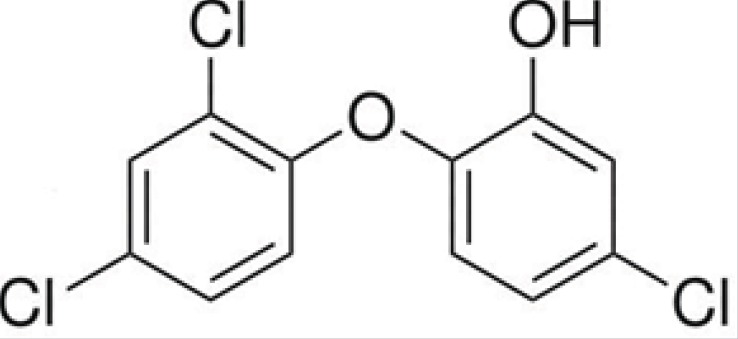
Chemical structure of Triclosan

The chemical structure of triclosan is shown in Figure 1.

 The *in-vitro *antimycoplasmal activities of triclosan, GAT, MXF, LVX, SPX, CIP, ENR and NOR were determined by using the standard two-fold dilution method following the aforementioned defined conditions. There are no universally accepted standards for mycoplasma susceptibility testing or specific MIC breakpoints (24, 25). However, some inference regarding the meaning of the obtained MICs can be made based on breakpoints established for other bacteria and the achievable concentrations. Generally speaking, if the MIC is ≤ 1.00 μg/mL, the drug may potentially be active against mycoplasma (26). Thus, the antimycoplasmal susceptibility testing results showed that *M. mycoides *subsp *Capri *Y-goat, *M. gallisepticum *BG44T, *M. gallisepticum *PG31 and *M. bovis *PG1 were all sensitive to the fluoroquinolones, with MICs ranging from 0.0150 μg/mL to 1.00 μg/mL. The only exception was that the MIC of NOR against *M. bovis *PG1 was 2.00 μg/mL (Table 1). Nevertheless, the clinical isolate, *M. bovis *8421, was resistant to all the tested fluoroquinolones, with MICs ranging from 2.00 μg/mL to 64.0 μg/mL. The MICs of triclosan against *M. mycoides subsp. Capri *Y-goat, *M. gallisepticum *BG44T, *M. gallisepticum *PG31 and *M. bovis *PG1 ranged from 16.0 to 32.0 μg/mL, while the MIC of triclosan against the clinical isolate, fluoroquinolones-resistant *M. bovis *8421, was 64.0 μg/mL. In addition, the experiments evaluating NaOH or DMSO in the solution demonstrated that the minute concentrations of the compounds were not significant and could be ignored. These results indicate that triclosan has potential antimycoplasmal activity.

**Table 1:** MICs and modulation factors of compounds for five mycoplasma species

**Fluoroquinolones**a	Speciesb	**MIC**c **(μg/mL)**		**Concentration of triclosan as modulator**d **(μg/mL)**	**Modulation factor**e **(fluoroquinolones)**	**FICI**	**Interpretation**
		**fluoroquinolones**	**triclosan**				
**GAT**	Y-goat	0.0150	16.0	4.00	8	0.375	SYN
	BG44T	0.0150	32.0	8.00	4	0.500	SYN
	PG31	0.0150	32.0	8.00	8	0.375	SYN
	PG1	0.0300	32.0	8.00	4	0.500	SYN
	8421	2.00	64.0	16.0	4	0.500	SYN
**MXF**	Y-goat	0.0150	16.0	4.00	4	0.500	SYN
	BG44T	0.0150	32.0	8.00	8	0.375	SYN
	PG31	0.0150	32.0	8.00	4	0.500	SYN
	PG1	0.0300	32.0	8.00	16	0.350	SYN
	8421	2.00	64.0	16.0	4	0.500	SYN
**LVX**	Y-goat	0.125	16.0	8.00	4	0.750	IND
	BG44T	0.0600	32.0	4.00	2	0.625	IND
	PG31	0.0600	32.0	16.0	4	0.750	IND
	PG1	0.125	32.0	16.0	64	0.516	IND
	8421	2.00	64.0	32.0	8	0.625	IND
**SPX**	Y-goat	0.0150	16.0	4.00	4	0.500	SYN
	BG44T	0.0150	32.0	8.00	4	0.500	SYN
	PG31	0.0150	32.0	8.00	4	0.500	SYN
	PG1	0.0600	32.0	16.0	8	0.625	IND
	8421	4.00	64.0	32.0	16	0.563	IND
**CIP**	Y-goat	0.125	16.0	8.00	4	0.750	IND
	BG44T	0.125	32.0	16.0	4	0.750	IND
	PG31	0.125	32.0	8.00	16	0.306	SYN
	PG1	0.250	32.0	8.00	2	0.750	IND
	8421	8.00	64.0	16.0	8	0.375	SYN
**EFX**	Y-goat	0.125	16.0	4.00	8	0.375	SYN
	BG44T	0.030	32.0	8.00	16	0.313	SYN
	PG31	0.030	32.0	8.00	8	0.375	SYN
	PG1	0.250	32.0	8.00	8	0.375	SYN
	8421	8.00	64.0	16.0	32	0.281	SYN
**NOR**	Y-goat	1.00	16.0	8.00	64	0.615	IND
	BG44T	1.00	32.0	8.00	2	0.750	IND
	PG31	1.00	32.0	16.0	4	0.750	IND
	PG1	2.00	32.0	16.0	4	0.750	IND
	8421	64.0	64.0	8.00	2	0.625	IND

aGAT = gatifloxacin; MXF = moxifloxacin; LVX = levofloxacin; SPX = sparfloxacin; CIP = ciprofloxacin; ENR = enrofloxacin; NOR = norfloxacin. bY-goat = *M. mycoides subsp Capri *Y-goat; BG44T = *M. gallisepticum *BG44T; PG31 = *M. gallisepticum *PG31; PG1 = *M. bovis *PG1; 8421 = *M. bovis *8421. cMICs of triclosan and fluoroquinolones were tested alone. dtriclosan served as a modulator. eModulation factor = MIC (fluoroquinolones)/MIC (fluoroquinolones + modulator).

*Drug susceptibility in combination*

Triclosan was further evaluated for its synergistic interaction with seven tested fluoroquinolones against mycoplasma by an MF and an FICI model. The MF and FICI values of triclosan, combined with fluoroquinolones, were calculated. The methods used to calculate these values are described in the materials and methods section. For all of the fluoroquinolones, triclosan was a great modulator. On average, it could lead to a 2 to 64-fold decrease in the MICs of fluoroquinolones (Table 1), at certain concentrations, for varied species. FICI values ranged from 0.281 to 0.750 (Table 2), indicating either synergism or indifference. For the five mycoplasma species, the addition of triclosan to GAT, MFX and EFX displayed MFs of 4-8, 4-16, and 8-32, respectively. The FICI values were 0.375-0.500, 0.350-0.500, and 0.281-0.375, respectively. These values revealed synergistic effects. For these same tested species, the combination treatments of triclosan with LVX, SPX, CIP or NOR displayed MFs of 2-64, 4-16, 2-16, and 2-64, respectively, with FICI values ranging from 0.516 to 0.750, 0.500 to 0.625, 0.306 to 0.750, and 0.615 to 0.750, respectively. These results indicated either synergistic activity or indifference. Moreover, the MICs of triclosan were decreased 2- to 8-fold when used in combination with fluoroquinolones. Interestingly, no antagonism was noted in any species when combination therapies were tested. As the MF and FICI values suggest, triclosan may be a potential inhibitor of mycoplasma and is worthy of further study.

**Table 2:** Combination testing with Triclosan plus antimycoplasmal drugs against Mycoplasma isolates.

**Combination of drugs**	**Species**	**MIC (μg/mL)**		**FICI**		**Interpretation**a
		**Individual**	**Combination**	**Individual**	**Combinatio**	
**Triclosan/GAT** **Triclosan/MXF** **Triclosan/LVX** **Triclosan/SPX** **Triclosan/CIP** **Triclosan/EFX** **Triclosan/NOR**	Y-goat	16.0/0.0150	4.00/0.00150	0.25/0.125	0.375	SYN
	BG44T	32.0/0.0150	8.00/0.00300	0.25/0.250	0.500	SYN
	PG31	32.0/0.0150	8.00/0.00150	0.25/0.125	0.375	SYN
	PG1	32.0/0.0300	8.00/0.00750	0.25/0.250	0.500	SYN
	8421	64.0/2.00	16.0/0.500	0.25/0.250	0.500	SYN
	Y-goat	16.0/0.0150	4.00/0.00300	0.25/0.250	0.500	SYN
	BG44T	32.0/0.0150	8.00/0.00150	0.25/0.125	0.375	SYN
	PG31	32.0/0.0150	8.00/0.00300	0.25/0.250	0.500	SYN
	PG1	32.0/0.0300	8.00/0.00300	0.25/0.100	0.350	SYN
	8421	64.0/2.00	16.0/0.500	0.25/0.250	0.500	SYN
	Y-goat	16.0/0.125	8.00/0.0300	0.50/0.250	0.750	IND
	BG44T	32.0/0.0600	4.00/0.0300	0.125/0.50	0.625	IND
	PG31	32.0/0.0600	16.0/0.0150	0.50/0.250	0.750	IND
	PG1	32.0/0.125	16.0/0.00150	0.50/0.016	0.516	IND
	8421	64.0/2.00	32.0/0.250	0.50/0.125	0.625	IND
	Y-goat	16.0/0.0150	4.00/0.00300	0.25/0.250	0.500	SYN
	BG44T	32.0/0.0150	8.00/0.00300	0.25/0.250	0.500	SYN
	PG31	32.0/0.0150	8.00/0.00300	0.25/0.250	0.500	SYN
	PG1	32.0/0.0600	16.0/0.00700	0.50/0.125	0.625	IND
	8421	64.0/4.00	32.0/0.250	0.50/0.063	0.563	IND
	Y-goat	16.0/0.125	8.00/0.0300	0.50/0.250	0.750	IND
	BG44T	32.0/0.125	16.0/0.0300	0.50/0.250	0.750	IND
	PG31	32.0/0.125	8.00/0.00700	0.25/0.056	0.306	SYN
	PG1	32.0/0.250	8.00/0.125	0.25/0.500	0.750	IND
	8421	64.0/8.00	16.0/1.00	0.25/0.125	0.375	SYN
	Y-goat	16.0/0.125	4.00/0.0150	0.25/0.125	0.375	SYN
	BG44T	32.0/0.030	8.00/0.0015	0.25/0.063	0.313	SYN
	PG31	32.0/0.030	8.00/0.0030	0.25/0.125	0.375	SYN
	PG1	32.0/0.250	8.00/0.0300	0.25/0.125	0.375	SYN
	8421	64.0/8.00	16.0/0.250	0.25/0.031	0.281	SYN
	Y-goat	16.0/1.00	8.00/0.0150	0.50/0.015	0.615	IND
	BG44T	32.0/1.00	8.00/0.500	0.25/0.500	0.750	IND
	PG31	32.0/1.00	16.0/0.250	0.50/0.250	0.750	IND
	PG1	32.0/2.00	16.0/0.500	0.50/0.250	0.750	IND
	8421	64.0/64.0	8.00/32.0	0.125/0.50	0.625	IND

a SYN, synergism; IND, indifference. For the FIC index model, synergism was defined as a FICI of ≤ 0.5, and indifference (*i.e*., no interaction) as a FICI of > 0.5 to 4

## Discussion

Mycoplasmas are important livestock and avian pathogens that cause respiratory and joint diseases which result in world-wide economic losses to livestock and poultry industries (27). The pathogen also causes a variety of clinical infections in humans, predominantly in the respiratory and/or urogenital tract, commonly causing tracheobronchitis (28). New and alternative antimicrobial substances should be urgently investigated. The development of drug-resistant mycoplasma species, which are resistant against commonly used antimicrobials, such as macrolides, lincomycin and streptogramin B (29), is concerning. Fluoroquinolones have become important therapeutic alternatives for the treatment of diseases and infections caused by a wide variety of microorganisms, including mycoplasma (28). In the present study, we investigated the antimycoplasmal properties of fluoroquinolones against four standard strains, and significant inhibiting activity was observed. In contrast, relatively weaker antimycoplasmal activity was shown against the clinical isolate *M. bovis *8421. MICs for this isolate ranged from 2.00 to 64.0 μg/mL using different fluoroquinolones, which is higher than the breakpoint of 1.00 μg/mL defined in a previous study (26). Thus, this clinical isolate should be regarded as resistant to fluoroquinolones. Notably, cross-resistance among the various fluoroquinolones is well documented (20, 30).

In this study, we investigated the *in-vitro *antimycoplasmal activity of triclosan and its synergistic interactions with fluoroquinolones against mycoplasma. We found that triclosan alone has potential antimycoplasmal properties against both fluoroquinolones-sensitive and fluoroquinolones-resistant strains, with MICs of 16.0 μg/mL, 32.0 μg/mL ,32.0 μg/mL, 64.0 μg/mL, and 64.0 μg/mL, for *M. mycoides subsp. Capri *Y-goat, *M. gallisepticum *BG44T, *M. gallisepticum *PG31, *M. bovis *PG1 and *M. bovis *8421, respectively. Particularly, for the combinations of triclosan with GAT, MXF, SPX, CIP, ENR, and NOR, the MICs of triclosan against all the tested species were decreased to less than 16.0 μg/mL. For the triclosan/LVX combination, the MICs of triclosan were 8.00 μg/mL, 4.00 μg/mL, 16.0 μg/mL, 16.0 μg/mL, and 32.0 μg/mL, respectively, against all strains. These values were decreased significantly, despite revealing weak synergism or indifference. In addition, the MICs of fluoroquinolones were decreased 2- to 64-fold by triclosan with FICI, ranging from 0.281 to 0.750, demonstrating a great deal of either synergism or indifference. Generally speaking, combinations of triclosan and fluoroquinolones not only exhibit excellent synergistic antimycoplasmal activity, but the combination of these drugs allows practitioners to lower the normal dosages of the fluoroquinolones used, which may help to prevent fluoroquinolones resistance within the mycoplasma population. To the best of our knowledge, it is the first time that the antimycoplasmal properties of triclosan, in combination with fluoroquinolones, have been evaluated against mycoplasma species.

Previous findings have shown that triclosan is not a carcinogen, mutagen, or teratogen (9, 11). Results of toxicology studies indicate that triclosan and its metabolites are well tolerated by a variety of livestock, as well as humans (9, 10, 31). Moreover, there have been no reports of triclosan-resistant microbes in the wild, although *Escherichia coli *and *Sphingosamine *strains have been selected for resistance *in-vitro *in the laboratory (11). Therefore, triclosan, which has the properties of broad spectrum antibiotic, non-toxic, absent of drug-resistance, and highly efficient antimicrobial activity, would be a remarkable modulator in combination with fluoroquinolones.

In conclusion, our study shows that triclosan has potential antimycoplasmal properties against fluoroquinolones-sensitive and fluoroquinolones-resistant mycoplasmas. Triclosan also possesses favorable synergistic activity or indifferent effects *in-vitro *with fluoroquinolones against mycoplasma species. These results are preliminary and further investigations are needed before triclosan could be used as a combination therapy *in-vivo*. Additionally, further analyses are needed to evaluate the underlying antimycoplasmal mechanism of triclosan when combined with fluoroquinolones.
